# Computer-Aided Ankle Ligament Injury Diagnosis from Magnetic Resonance Images Using Machine Learning Techniques

**DOI:** 10.3390/s23031565

**Published:** 2023-02-01

**Authors:** Rodrigo S. Astolfi, Daniel S. da Silva, Ingrid S. Guedes, Caio S. Nascimento, Robertas Damaševičius, Senthil K. Jagatheesaperumal, Victor Hugo C. de Albuquerque, José Alberto D. Leite

**Affiliations:** 1 Graduate Program in Surgery, Federal University of Ceará, Fortaleza 60455-970, CE, Brazil; 2 Department of Teleinformatics Engineering, Federal University of Ceará, Fortaleza 60455-970, CE, Brazil; 3 Department of Software Engineering, Kaunas University of Technology, 51368 Kaunas, Lithuania; 4 Department of Electronics and Communication Engineering, Mepco Schlenk Engineering College, Sivakasi 626005, TN, India

**Keywords:** ankle ligament injury, MRI, data augmentation, feature extraction

## Abstract

Ankle injuries caused by the Anterior Talofibular Ligament (ATFL) are the most common type of injury. Thus, finding new ways to analyze these injuries through novel technologies is critical for assisting medical diagnosis and, as a result, reducing the subjectivity of this process. As a result, the purpose of this study is to compare the ability of specialists to diagnose lateral tibial tuberosity advancement (LTTA) injury using computer vision analysis on magnetic resonance imaging (MRI). The experiments were carried out on a database obtained from the Vue PACS–Carestream software, which contained 132 images of ATFL and normal (healthy) ankles. Because there were only a few images, image augmentation techniques was used to increase the number of images in the database. Following that, various feature extraction algorithms (GLCM, LBP, and HU invariant moments) and classifiers such as Multi-Layer Perceptron (MLP), Support Vector Machine (SVM), k-Nearest Neighbors (kNN), and Random Forest (RF) were used. Based on the results from this analysis, for cases that lack clear morphologies, the method delivers a hit rate of 85.03% with an increase of 22% over the human expert-based analysis.

## 1. Introduction

Chronic ankle instability is mostly caused by ankle strain and it develops mostly as anterior talofibular ligament (ATFL) injury [1]. By observing its progressive nature, it may also provoke a chain of injuries on tendons and cartilage, among them a few of which are irreversible, where the early diagnosis and treatment of chronic ankle instability may reduce morbidity [2]. ATFL injury is considered as one of the most frequent types of ankle injury [3]. Lateral ligament injuries usually occur during plantar flexion and inversion, which is the position of maximum stress on the ATFL ligament, which is the most common ligament to tear, and may occur during inversion injury [4].

Among sedentary patients, instability complaints can be too late for making subsidiary tests, which are crucial for early treatment. Magnetic resonance imaging (MRI) provides optimal visualization of morphological changes in ankle ligament structures [5,6,7,8], however, the present set of literature lacks an accurate definition of a normal ATFL morphology [9,10,11,12]. Moreover, low accuracy is also observed in many studies [12,13,14] which could be due to the fact that it is a static exam used to study a structure that performs dynamic joint control. Further, it can potentially produce false negatives for ligament lesions, which would occur if the ligament was subjected to a yo-yo effect [11,13,15,16,17,18]. While staying at rest, it would exhibit a normal morphological structure, but leads to increased fiber elasticity, caused by a bad local collagen quality or quantity [19]. The prediction on the dynamic behavior of the ligament is possible even in a static exam such as MRI since this exam generates a good definition of the ultrastructure of tissues [1,17,20,21]. However, this detailed characterization is not possible in medical diagnosis due to its lack of precision, however, it could be addressed with the aid of computer vision [13,22].

In view of this, several studies have been carried out with the aim of proposing Computer Aided Design (CAD)-based medical diagnosis systems for various fields of medicine such as the diagnosis of Parkinson’s disease [23] and the Alzheimer’s disease [24], recognition and detection of atrial fibrillation [25,26], virtual nasal endoscopy system [27], lung nodule detection [28], oral cancer classification [29], breast cancer detection [30,31], as well as ankle prostheses and diseases [32,33,34], and many more [35,36,37,38].

This study aims to compare the human analysis of morphological changes in ATFL with computer vision for extraction of image characteristics and analyze them in comparison with the patient’s clinical presentation.

The principle contributions of this study are listed below:

- Stratify the possibilities of morphological variations on the ligament and its correction with ankle instability;
- Compare the ability for diagnosis by the magnetic resonance of different evaluators;
- To develop a method for extracting and classifying ankle ligaments to aid medical management;
- To compare and analyze different feature extraction techniques;
- Validate the results through statistical evaluations;
- To compare and analyze human diagnostic capability with software-based capability.

## 2. Materials and Methods

In this section, the stages of patient selection and the application of the proposed method will be presented, by addressing the forms of selection of the area of the ligament, through the feature extraction methods. Subsequently, the classification strategies chosen for analysis will be highlighted along with their core operational principles in automated ankle ligament injury diagnosis.

### 2.1. Ethical Statements

All subjects gave their informed consent for inclusion before they participated in the study. The study was conducted in accordance with the Declaration of Helsinki, and the protocol was approved by the Institutional Research Ethics Committee Medical School of the Federal University of Ceará, Fortaleza, Ceará, Brazil under CAE:54356421.5.0000.5043 in December 2021.

### 2.2. Patient Selection

In this work, according to the criteria adopted by the International Ankle Consortium (IAC) as shown in Table 1 [22], patients were categorized into two groups: with or without Chronic Ankle Instability (CAI). The evaluation was carried out with the consideration of the following criteria compiled from the literature, as well as a foot and ankle surgeon with 10 years of experience and an orthopedist with 40 years of experience.

This is a prospective study done at a postgraduate program in surgery at Universidade Federal do Ceará (UFC) conducted between August 2021 and January 2022 using morphological analysis of the ATFL in MRI examinations, in a T2-weighted axial section, from 321 patients. Tests were done for diagnostic purposes related and not related to ankle instability. Exclusion criteria included were patients with ankle lesions and without a history of fracture, surgery, or anatomical deformities [20,39]. ATFL usually consists of two fiber bundles, and seldom has three bands, as reported in previous studies [40,41]. All these bundles are usually present in the 0.57 mm anterior fibular margin from the tip of the fibula [42]. In this area, the cut with the best visualization having the origin and insertion of the ligament was selected for analysis by author number 1, with the protocol considered for analysis with T2 axial cuts was TR=3512 m, TE=58.56, FOV=66×24 cm, and ST=4 mm.

### 2.3. Computational Characterization of the ATFL

**Figure 1:**
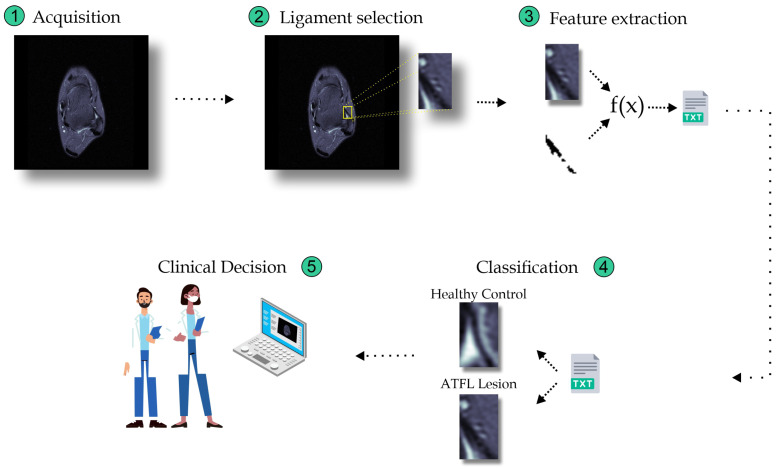
Sequence of stages involved in the clinical decision making of ankle ligament injury from the MRI images.

With the image added, an interface is opened to select the region of the ligament. With the selection of the ligament area performed, the feature extractors are applied, performing all possible combinations, individually, in pairs, in trios, and all together. Finally, these images are classified into ATFL Lesion and Healthy Control. Figure 1 highlights the general sequence of stages involved in clinical decision-making from the acquisition of ankle ligament MRI images.

### 2.4. Description of the Database

The MRI images considered for this study are obtained from the Vue PACS–Carestream program in DICOM format, as axial T2-weighted slices at the ATFL level. Among them, 17 images are with ATFL diagnosis and another 17 images are with the healthy diagnosis kind. For increasing the number of images in the database, the data augmentation technique was applied. The image transformations used were shear, rotation, horizontal translation (x-axis), and vertical translation (y-axis), with 10${}^{\circ }$, 10${}^{\circ }$, 5 ${}^{\circ }$ and 5${}^{\circ }$ values, respectively. For each image, the type of transformation is randomly selected, incrementing three times. Thus, the final database will contain the images after the image transformation process together with the original images.

### 2.5. Data Extraction

To perform feature extraction from ankle computed tomography images, the following methods were used.

#### 2.5.1. Gray Level Co-Occurrence Matrix (GLCM)

GLCM is used for analyzing pixels in the images, using texture calculations to determine the level of gray in the second order. It represents the relative frequencies of a pair of shades of gray, that are present at a certain distance and angle. The elements of GLCM are the relative frequencies $P_{ij}$ of the presence in the image neighboring points with bright points $I_{i}$ and $I_{j}$ located at a distance *d* from each other in one from four angular direction {0,45,90,135} degrees [43]. Six features are extracted through the GLCM technique, namely: contrast, dissimilarity, homogeneity, energy, correlation, and angular second moment [44,45,46].

#### 2.5.2. Local Binary Patterns (LBP)

LBP is a method that considers eight neighbors of a pixel and labels the pixels in the image with the neighborhood threshold of each one, treating the result as a binary number [47]. LBP uses a local area pattern to describe the texture, where each pixel is marked by a code value formed by the original texture from the local neighborhood that best matches it. By applying the LBP operator to each pixel of the image, we can construct a histogram in which each LBP code corresponds to a separate column. MRI images can be viewed as a set of all sorts of local features that are well described using local binary patterns. To take into account information about their location in the image, the image is divided into sub-regions, wherein each LBP histogram is calculated. By concatenating these histograms, a general histogram can be obtained that takes into account both local and global features of the image.

#### 2.5.3. Hounsfield Unit Invariant Moments

The Hounsfield scale is a quantitative scale for the radiodensity of MRI and other similar type images. The scale of Hounsfield units (HU) is a linear lesion scale relation to dispersed water, the bulk density of which was taken as 0 HU. By using HU, one can easily filter images, removing all pixel values that differ from the one we are interested in, in a range by more than the specified threshold. Thus, one can select all pixels in the image, on which, according to the Hounsfield scale bones are displayed.

The HU invariant moment is a method used to obtain the characteristics of digital objects, such as position, axis rotation, and changes in scale, reducing errors in recognition or identification [48]. In [49], seven functions were defined as nonlinear representation at regular moments that are invariant to rotation, scale, and translation.

#### 2.5.4. Dimensional Characteristics (DC)

In order to analyze some relevant information regarding the computed tomography of the ankle, measures such as ligament length, total area, the standard deviation of the ligament, and the standard deviation of the selected area were calculated, which analyzes both the ligament and the neighboring regions.

### 2.6. Classification Methods

To classify the data extracted by GLCM, LBP, Invariant Moments of HU, and dimensional characteristics, the following set of classifiers were used for analysis.

#### 2.6.1. Multi Layer Perceptron (MLP)

MLP is a supervised learning algorithm, of the type feedforward artificial neural network (ANN) that consists of at least three layers. Here, the input layer is used to collect the input signal, and the output layer provides a decision. The set of layers between the input and output layers are treated as a hidden layer [50].

This classifier uses a backpropagation algorithm to perform data training, thus each layer is connected by weight, threshold, and transfer function to transfer data from the front to the back to the output layer. If the error between output layer data and the known data does not match the target error, the layer weights and limits are adjusted backward according to the training algorithm until the error converges to the desired limit [51].

#### 2.6.2. Support Vector Machine (SVM)

SVM is a supervised learning algorithm that employs structural risk minimization from statistical learning theory [52]. SVM can be used both on linearly separable and non-linearly separable data. In cases where the data are linearly separable, SVM builds an optimal hyperplane that separates two different groups of feature vectors with a maximum margin. In certain cases, where the data are not linearly separable, SVM maps its input into a high-dimensional feature space by applying a kernel function [53].

#### 2.6.3. Random Forest (RF)

The RF algorithm is one of a supervised learning method that includes a series of tree predictors, where each tree is based on the values of a randomly sampled vector with the same distribution across all trees in the forest. Thus, the results of each of these trees are calculated separately and then combined to provide a favorable prediction [54,55]. RF is an enhanced version of the decision tree algorithm, considering that the classification capacity of a single tree may be small. When a large number of decision trees are generated at random, the most likely categorization of the test samples may be determined based on the classification results of each tree [56].

#### 2.6.4. k-Nearest Neighbors (k-NN)

The k-NN algorithm is one of a non-parametric method, which is used for classification that checks the classes of a chosen number of training data samples surrounding a test data sample. Further, in order to make a prediction of which class the training data sample will belong to, it finds similarities in the data on every test observation [57,58]. The input consists of the *k*-training samples, while the classification is done by voting the class of each neighboring data point by assigning it to the class. So, the samples find their place at *k*-nearest neighbors in the class, so that when completed, the output is classified, and its association indicates which of the data points belongs to which class.

## 3. Experimental Setup and Performance Metrics

In this section, the experimental setup chosen for the deployment of the MRI analysis of the classifiers, as well as the validation metrics with the chosen configurations are presented. Subsequently, the medical analysis strategy used in this study is elaborated. As the most recent studies analyzed between 100 and 600 patients, and most of them classified the ligament in a more simplified fashion as normal ligament or altered ligament [14,17], this study made the difference between a normal, abnormal, and absent ligament, and so a larger sample was chosen.

The dataset obtained through the extractors used was split using K-fold Cross-Validation with 20 fields. Hyper-parameters were tuned using a grid search. The SVM parameters are the $\gamma \in [2^{-15},2^{-1}]$ and $C\in [2^{-5},2^{5}]$. The RF parameter is the number of trees in the forest ∈ [50:500]. The MLP parameters were the number of hidden layers ∈ [1:5], the number of neurons in hidden layer ∈ [50:500], α ∈ [00001.1], and learning rate ∈ [00001,0.9999]. In K-NN, the number of neighbors ∈[3,10] and leaf size ∈[10,50].

The experiments were executed on a PC with Windows 11 operating system, 8-core i7 11800H 4.6 GHz CPU, 16 Gb of DDR4 RAM, 3200 MHz, and NVIDIA Geforce RTX 3060, 6 GB GDDR6 graphics card.

### 3.1. Validation Metrics

**Figure 2:**
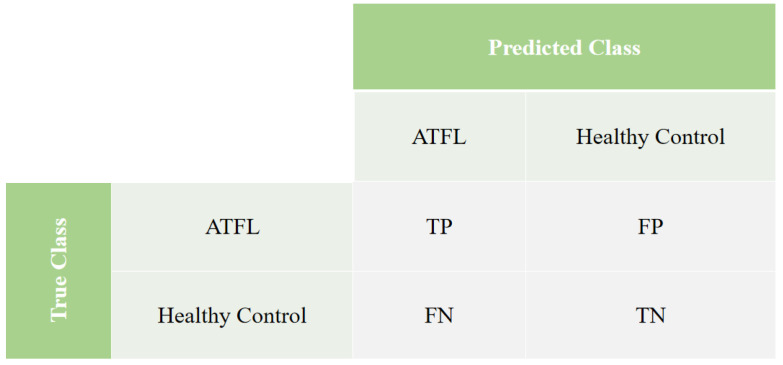
The confusion matrix highlights the validation metrics chosen for experimentation and analysis.

To assess the performance, a confusion matrix was used employing the concepts of True Positive (TP), False Positive (FP), True Negative (TN), and False Negative (FN), as shown in Figure 2.

Thus, we have the definitions of TP, FP, TN, and FN below:

- **True Positive (TP):** The TP occurs when considering the real dataset, where the ATFL class was predicted correctly as the ATFL class;
- **True Negative (TN):** The TN occurs when considering the actual dataset, where the healthy control class was correctly predicted as the healthy control class;
- **False Negative (FN):** The FN occurs when considering the real set of data, where the class that is sought to be predicted was incorrectly predicted. This happens, when it was supposed to be ATFL and was classified as a healthy control;
- **False Positive (FP):** The FP occurs considering the real set of data, where the class that is sought to be predicted was incorrectly predicted. This happens, when it was supposed to be healthy control and was classified as ATFL.

Considering the confusion matrix, five metrics were used in order to evaluate the results, namely: Accuracy (AccGlobal), f1-score (F1score), ATFL class hit rate (ATFL), and class hit rate Healthy Control (HealthyControl).

- **Accuracy:** Refers to the global hit probability, which is the measure of general hit rate considering the two analyzed classes, considering errors and hits.
  $$AccGlobal=\frac{TP+TN}{TP+TN+FP+FN}\times 100\%$$
- **F1-score:** Refers to the harmonic mean between accuracy and recall. It is often used to evaluate unbalanced bases.
  $$F1-score=2\times \frac{Precision*Recall}{Precision+Recall}\times 100\%$$
- **ATFL class hit rate (ATFL):** Refers to the probability that a patient who has a positive diagnosis for ATFL actually has ATFL.
  $$ATFL=\frac{TP}{TP+FP}\times 100\%$$
- **Healthy control class hit rate (HealthyControl):** Refers to the probability of a patient who has a negative diagnosis for ATFL, that is, a patient from the healthy control class and that does not have ATFL.
  $$HealthyControl=\frac{TN}{TN+FN}\times 100\%$$

### 3.2. Medical Analysis

**Figure 3:**
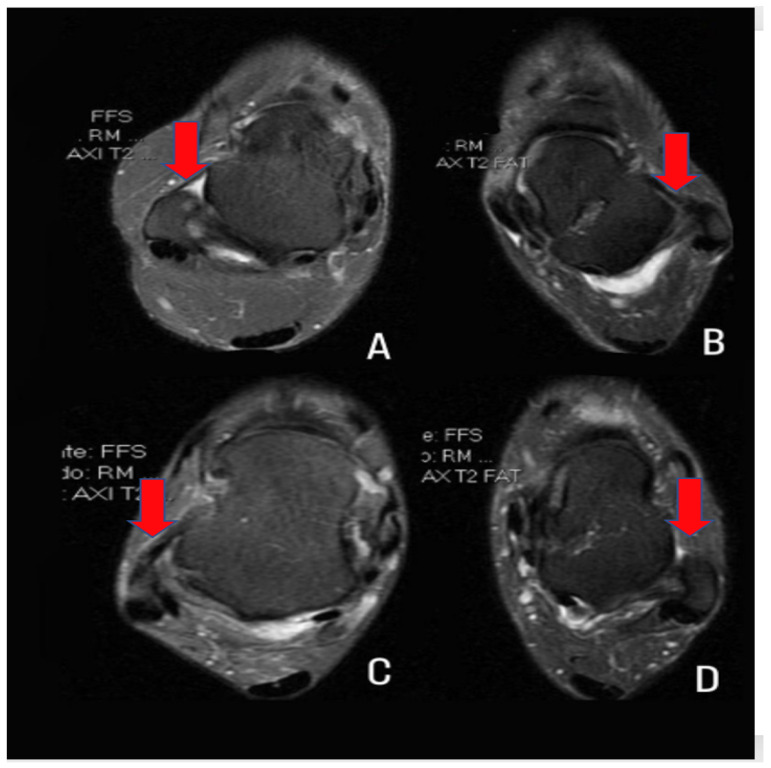
MRI of the ankle in a T2-weighted axial section at the height where the talus is most visible for ATFL analysis. (**A**): normal ligament (rectilinear and homogeneous); (**B**): abnormal ligament due to altered signal; (**C**): abnormal ligament due to altered contours; and (**D**): absent ligament.

For human analysis, ATFL was assessed by two examiners (one foot and ankle surgeon, both with rich experience of 10 and 40 years, respectively, in their corresponding fields) to address potential sources of bias, with comparative intra and interobserver analysis using the intraclass correlation coefficient (ICC). ATFL thickness was measured in T2-weighted axial sections, selecting the section where the highest ligament thickness was obtained at its midpoint. In addition to thickness, the ligaments were classified as normal, abnormal (altered signal or contour), or absent (Figure 3).

IBM SPSS Statistics (IBM, Armonk, NY, USA) was used for calculations. The characteristics of the study population were analyzed using the *t*-test with equal variables (after analysis with the F-test) and a fixed alpha of 0.05 for all the comparisons. The following variables were analyzed: minimum, maximum, average, standard deviation, and Student’s *t*-test at a 95% confidence interval of the ligament thickness obtained in addition to the intraclass correlation coefficient (0.83). Moreover, the minimum, maximum, average, and standard deviation of the participant’s age was also calculated to analyze possible study limitations.

## 4. Results and Discussion

In this section, the results obtained from the proposed methodology in association with the state-of-the-art methods used in this work will be discussed, both for human and computational analysis.

### 4.1. Human Analysis

**Figure 4:**
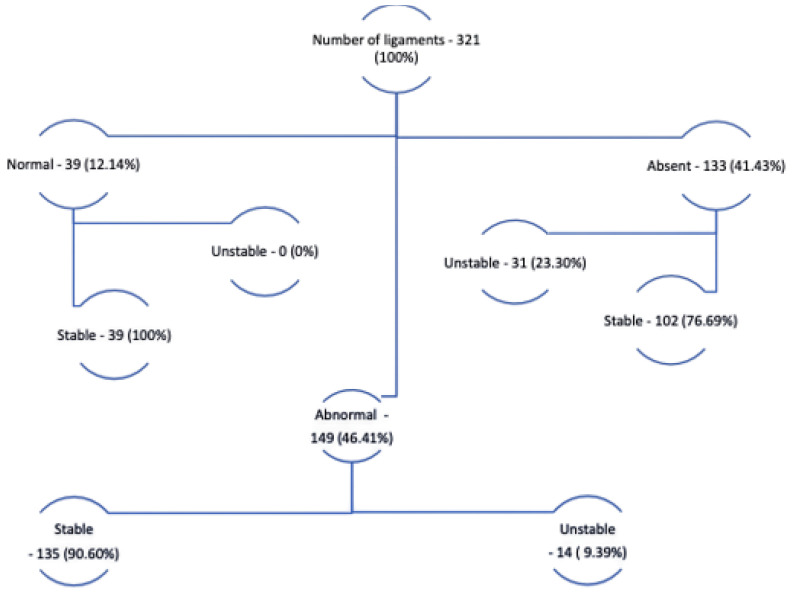
Distribution of normal, abnormal, and absent ATFL and CAI.

A total of 321 patients were included for the study, among them 220 and 101 were women and men, respectively, aged between 18 and 81 years, with an average of 33.5 years. After analysis, 276 patients were found to have stable ankles and 45 were diagnosed as unstable. An alpha of 0.830 was used for the statistical analysis of interrater reliability. The Intraclass Correlation Coefficient (ICC) was 0.710 and the 95% confidence interval exhibited a lower and upper limit of 0.627 and 0.777, respectively. The proportions between the morphological characteristics of the ligaments and CAI diagnosis are presented in Figure 4.

MRI sensitivity and specificity in diagnosing CAI were assessed in two situations, considering CAI when the ligament exhibited any morphological change or only CAI when the ligament was absent. The findings from these analysis are described in Table 2.

In this way, it is demonstrated that the most frequent morphology found for LFTA is not a rectilinear homogeneous ligament, since only 39 of the 321 ankles analyzed exhibited this morphology. Among the 282 ligaments with some morphological change, only 45 had a history of chronic ankle instability. This high prevalence of morphological ligament changes is likely explained by the high incidence of ankle strains and primarily instability.

Indeed, it is unlikely that an individual that walks on irregular terrain and engages in sports would not experience some ankle injury by adulthood, including a painless lesion such as in the case of instability. Our results indicate that despite being painless, ankle instability that occurs over time may cause morphological changes in the LFTA.

### 4.2. Computational Analysis

For each algorithm considered for the analysis, the feature extraction time is estimated, which could be inferred from Table 3.

Thus, it can be observed that the HU algorithm reached the shortest extraction time with 0.026 s, followed by the LBP with 0.121 s. The GLCM algorithm obtained the highest extraction time compared to the others, with a total of 2.219 s. For identification purposes, we defined representations for each combination of extraction algorithms, as shown in Table 4.

Thus, for each Set, the classification processes were carried out using the chosen set of algorithms in this study. The Table 5 highlights the results obtained with the obtained metrics from each of the classification algorithms. Although ACC Global verifies a general measure of accuracy, the important consideration for medical diagnosis is the accuracy of the ATFL, so the algorithm with the highest of accuracy in the ATFL will be considered to be most appropriate.

Analyzing the extractors individually from Set 1 to Set 4, the highest percentage of correct answers was obtained for Set 2, which represents the LBP extractor with the RF classifier, reaching 80.60% of Global Acc and 83.35% of ATFL. The observations from the extractors analyzed individually is that Set 3, that is, the HU extractor, was one of those that obtained the lowest percentages by considering the analysis among all the four classifiers. This situation is expected to be due to the shape data features extracted by the HU algorithm.

When analyzed in pairs, that is, from Set 5 to Set 10, the Set 5, GLCM with LBP, obtained the highest result with ATFL of 85.03% when classified with the RF algorithm. The lowest result found in this analysis was Set 6, GLCM with HU, and MLP classifier with ATFL 17.95%. The combinations of three extractors, from Set 11 to Set 14, with Set 12 and RF classifier obtained an ATFL percentage of 84.34%. The trio that obtained the lowest result was the trio from Set 11 and MLP classifier with ATFL of 9.25%.

In general, after vigorous analysis by considering all the sets, the ones that obtained the best results for the ATFL was Set 5 with the RF classifier. It is observed to be statistically similar, through the Wilcoxon test, with Set 12 and the RF classifier. It can be seen that the MLP classifier obtained the lowest success rates, whether in the ATFL class or in global accuracy, which could have been caused by the low number of images, unlike the RF classifier, which obtained the highest percentages of success.

**Figure 5:**
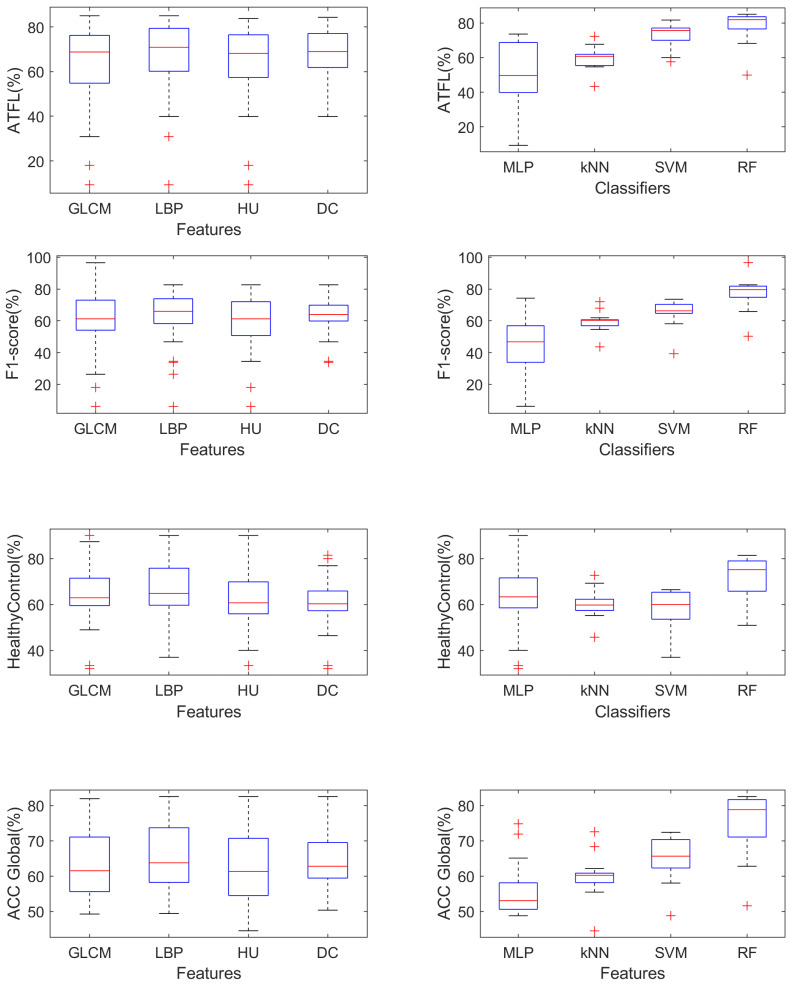
Comparison of mean values of performance measures for various combinations of feature sets and classifiers.

We summarize and compare the performance measures (ATFL, F1-score, HealthyControls, ACC Global) achieved using various combinations of feature extraction methods (GLCM, LBU, HU, DC) and classification methods (SMLP, kNN, SCM, RF) used in this study in Figure 5. Note that the selection between different feature sets has a small influence on performance measures, while more variability can be observed when selecting different classifiers.

**Figure 6:**
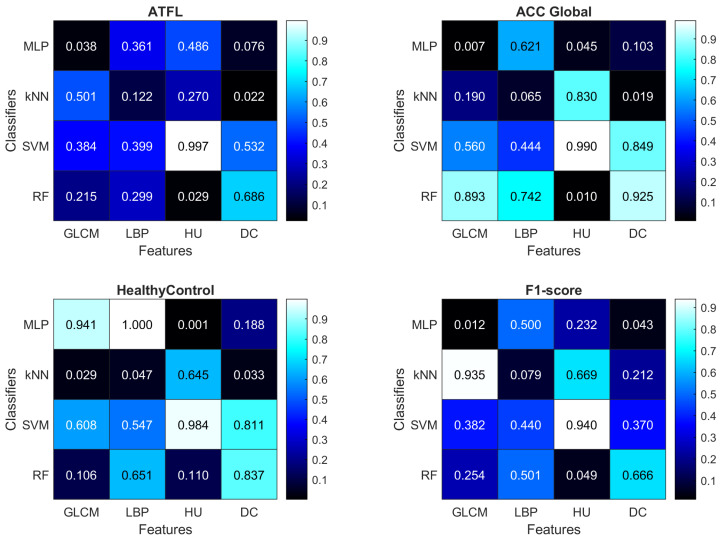
Results of the statistical *t*-test (*p*-values) for various combinations of feature sets and classifiers.

**Figure 7:**
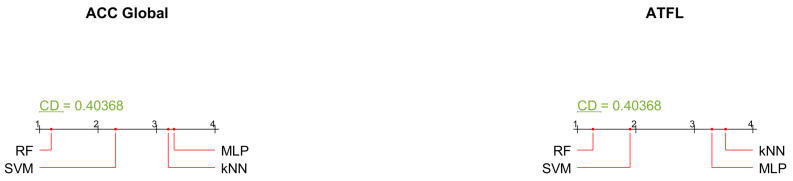

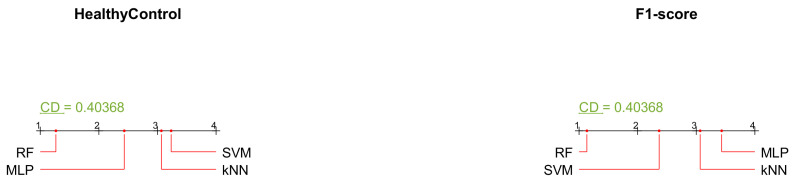
Critical Distance diagrams for performance metrics of the machine learning methods.

To analyze the performance differences between feature extraction and classification methods, we have adopted a two-sample *t*-test, which returns a decision on the null hypothesis that the data in both samples has equal means and equal but unknown variances. The 5% significance level was used. We compared the remaining results of all sets using the considered feature set (e.g., LBP) vs. all sets that are not using the considered feature set. The procedure was repeated for all combinations of feature sets and classifiers. The results are visualized as heatmaps in Figure 6, while the most notable cases are discussed below:

- Using the GLCM features improves the ACC Global measure when using the MLP classifier (p<0.01);
- Using the HU features improves the ACC Global measure when using the RF classifier (p<0.01), and the HealthyControl measure when using the MLP classifier (p<0.01);
- SVM is never able to outperform significantly (p<0.05) other classifiers;

When comparing more than two methods, a multiple test procedure is recommended. When the null hypothesis of equivalent performances for multiple methods is rejected, post hoc tests are used to identify the significantly different methods. The Nemenyi test is a post-hoc test that is performed after the Friedman test. The Nemenyi test is used to compare the relative performance of all classifiers evaluated in the study [59]. The differences in performance of various classifiers are compared to the value of Critical Distance (CD) obtained using the following equation.

(1) $$CD=q_{\alpha }\sqrt{\frac{m\left(m+1\right)}{6n}}$$

where *m* is the number of classifiers, *n* is the number of datasets, and *q* is based on the Nemenyi test’s studentized range statistics. The Nemenyi test can be easily understood using the critical distance diagrams shown in Figure 7. The results show the superiority of RF classifier results as compared to SVM, MLP, and kNN.

**Figure 8:**
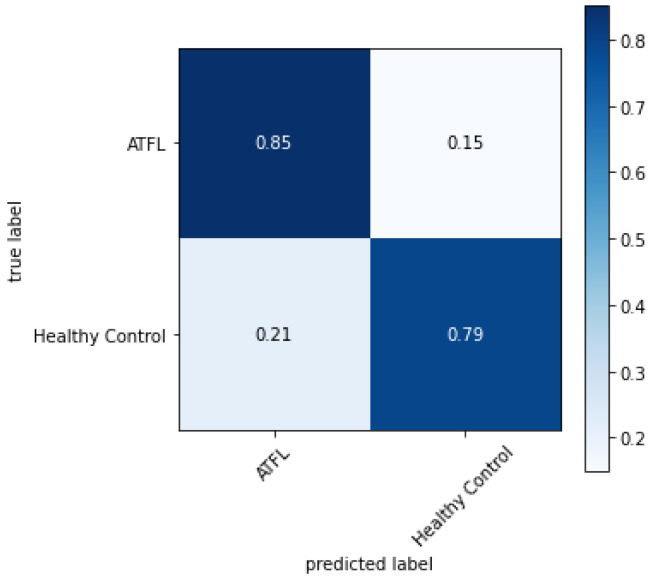
Confusion matrix obtained for the RF classifier with Set 5 chosen for analysis.

In Figure 8, the confusion matrix is presented for the highest percentage of correct ATFL, obtained by Set 5 with the RF classifier. A higher success rate is obtained in identifying ATFL. Further, consequently, a lower rate of false positives rate was 0.15, i.e., 15%, which may have been caused by the texture similarities of the normal and ATFL areas, as well as the failure to analyze geometric structures.

However, when analyzing the texture extractors together with the shape extractors, there is not a big difference in the percentage of correct predictions, as can be seen in Set 12, which was statistically equal to Set 5. Thus, other characteristics besides shapes and textures can contribute to the reduction of false positives.

Regarding the training and testing times of each classifier for each Set, the k-NN algorithm for Set 14 achieved the shortest training time with 0.00018 s. Moreover, the SVM algorithm for Set 2 obtained the shortest training time with 0.00019 s. Later on, it is observed that the longest training time was achieved by the SVM classifier, while the longest testing time was obtained by the RF algorithm. These times can be observed as shown in Table 6.

**Figure 9:**
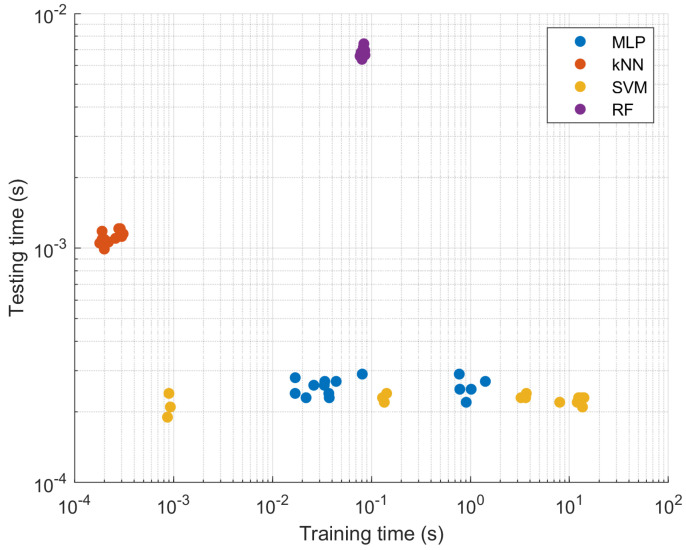
Comparison of classifiers based on training and testing times.

The comparison of classifiers based on training and testing times is presented in Figure 9. The lowest training times are achieved by kNN while the lowest testing time is reported by SVM. RF has the worst time performance both in terms of training time and testing time.

### 4.3. Comparative Evaluation between Human Analysis and Computational Analysis

Human assessment of ligament morphological quality is limited. As shown in this study, the sensitivity of the evaluation only increases when the ligament has a clear morphology, that is, when the ligament was completely intact or completely absent. When analyzing the MRI images of the ligaments that do not show clear morphologies, the diagnosis of the ligament injury status using the proposed ATFL method is 85.03%, while the specialist’s diagnosis is below 63%. Thus, a gain of approximately 22% in the hit rate can be observed when using the proposed computational method.

On the other hand, in other tissues such as the ACL, this difference was not observed as expected [60,61]; this may occur because the ACL is a larger ligament, surrounded by joint fluid that enhances its border. However, the ATFL considers the thickening of the joint capsule by having joint fluid on the joint face, but also considers the no fluid case to enhance ligament definition on the anterior face of the ligament.

## 5. Conclusions and Future Work

Medical analysis of the quality of the ATFL is limited, with ligament damage evident in the complete absence of the ligament or normality of ligament function when, in its perfect anatomy (parallel edges, homogeneous dark gray pattern in the ligament), morphological changes are present, such as alteration of the contour or homogeneity of the shade of gray, and the diagnosis becomes inaccurate. This study aimed to compare the human analysis of morphological changes in ATFL with computer vision strategy for image characteristics extraction and to compare it with the patient’s clinical presentation. The analyses were carried out by specialists and computational analysis using a computer vision method for ligament analysis. Using the proposed method can significantly increase the accuracy rate when analyzing images that do not have ligaments with perfect anatomy. From the analysis through these cases, the method reached a hit rate of 85.03% while the hit rate based on the expert analysis achieved a rate below 63%.

For future work, one might consider increasing the characterization of ankle instability by adding ultrasonography under the stress of the ligament to the clinical diagnosis that was made of the ligament injury, in addition to increasing the database with more patients in the ankle instability group. In computational matters, one can seek to reconstruct the 3D images to analyze the images in different ways and metrics such as volumetric analysis.

## Figures and Tables

**Table 1:** Inclusion criteria for patients.

Standard inclusion criteria endorsed by the international ankle consortium for enrolling patients who fall within the heterogeneous condition of chronic ankle instability in controlled research	
1. A history of at least one significant ankle sprain	
At least 12 months prior to study enrollment	Associated with inflammatory symptoms
Created at least one interrupted day of desired physical activity	Acute traumatic injury to the lateral ligament complex of the ankle joint as a result of excessive inversion of the rear foot or a combined plantar flexion and adduction of the foot. This usually results in some initial deficits of functional and disability.
2. A history of the previously injured ankle joint “giving way”, and/or recurrent sprain, and/or “feelings of instability”	
Subjects should report at least two episodes of giving way in the six months prior to study enrollment	Giving way: the recurring occurrence of uncontrolled and unpredictable bouts of excessive rear foot inversion that do not result in an acute lateral ankle injury.
Recurrent sprain: two or more sprains to the same ankle	Self-reported ankle instability confirmed with a validate ankle instability, a specific questionnaire using the associated cutoff score: Ankle Instability Instrument, answering yes to at least five yes/no question.
3. Foot and Ankle Outcome Score: score of <75% in three or more categories	

**Table 2:** MRI sensitivity, specificity, and accuracy for the diagnosis of CAI in three different situations: normal, abnormal, or absent ligament. In the normal ligament scenario, only a normal ATFL is considered a stable ankle and the MRI is negative for CAI, in the absent ligament scenario only absence is considered positive for CAI. In the abnormal ligament scenario, absent ligament cases were excluded and any other abnormality on ATFL was considered positive for CAI.

Metric Values, %	Normal Ligament	Absent Ligament	Abnormal Ligament
Sensitivity	100%	68%	100%
Specificity	16%	63%	22%
Accuracy	26%	63%	16%

**Table 3:** Extraction times for each algorithm in seconds.

Algorithm	Time (s)
GLCM	2.219
LBP	0.121
HU	0.026
DC	1.528

**Table 4:** Representation of the combinations of feature sets.

Algorithms	Representation
GLCM	Set 1
LBP	Set 2
HU	Set 3
DC	Set 4
GLCM + LBP	Set 5
GLCM + HU	Set 6
GLCM + DC	Set 7
LBP + HU	Set 8
LBP + DC	Set 9
HU + DC	Set 10
GLCM + HU + LBP	Set 11
GLCM + DC + LBP	Set 12
GLCM + HU + DC	Set 13
LBP + HU + DC	Set 14
GLCM + LBP + HU + DC	Set 15

**Table 5:** Detailed results of the evaluation metrics.

Algorithms	Metrics (%)	MLP	kNN	SVM	RF
Set 1	ACC Global	49.26 ± 2.12	55.73 ± 5.18	59.75 ± 5.14	70.60 ± 5.74
	ATFL	49.40 ± 43.60	54.70 ± 11.94	57.64 ± 13.49	75.98 ± 11.03
	HealthyControl	48.92 ± 44.98	56.79 ± 11.66	61.83 ± 14.96	65.29 ± 10.60
	F1-score	35.99 ± 30.36	54.62 ± 7.35	58.13 ± 7.06	96.50 ± 1.82
Set 2	ACC Global	74.87 ± 3.94	72.56 ± 7.51	58.90 ± 5.88	80.60 ± 4.84
	ATFL	73.28 ± 8.95	72.20 ± 12.38	81.69 ± 15.37	83.35 ± 6.98
	HealthyControl	76.39 ± 8.20	72.69 ± 10.19	36.94 ± 22.44	77.72 ± 9.70
	F1-score	74.21 ± 4.75	72.03 ± 8.65	66.05 ± 4.75	81.11 ± 4.45
Set 3	ACC Global	48.78 ± 1.11	44.51 ± 6.06	48.78 ± 1.11	51.58 ± 7.28
	ATFL	60.00 ± 48.98	43.40 ± 9.06	60.00 ± 48.98	49.78 ± 9.62
	HealthyControl	40.00 ± 48.98	45.69 ± 8.10	40.00 ± 48.98	53.36 ± 9.57
	F1-score	39.34 ± 32.12	43.49 ± 7.72	39.34 ± 32.12	50.35 ± 7.92
Set 4	ACC Global	62.80 ± 6.40	60.36 ± 5.91	65.85 ± 5.82	65.73 ± 6.38
	ATFL	65.55 ± 12.84	61.82 ± 11.46	77.98 ± 14.22	68.23 ± 13.68
	HealthyControl	60.19 ± 14.29	58.92 ± 8.96	53.70 ± 12.01	63.27 ± 9.19
	F1-score	63.32 ± 7.54	60.47 ± 7.72	69.00 ± 6.98	65.94 ± 8.38
Set 5	ACC Global	51.82 ± 4.74	58.29 ± 6.16	64.75 ± 6.09	81.95 ± 4.52
	ATFL	30.88 ± 38.94	54.96 ± 12.33	69.63 ± 10.70	**85.03 ± 7.10**
	HealthyControl	72.67 ± 38.65	61.72 ± 9.15	59.98 ± 10.15	78.92 ± 7.65
	F1-score	26.37 ± 28.20	56.11 ± 8.96	66.02 ± 6.53	82.35 ± 4.54
Set 6	ACC Global	53.04 ± 5.71	55.48 ± 6.97	65.24 ± 6.97	71.09 ± 4.89
	ATFL	17.95 ± 29.87	54.64 ± 12.58	70.03 ± 13.38	76.54 ± 8.30
	HealthyControl	87.30 ± 23.85	56.50 ± 10.91	60.35 ± 9.23	65.74 ± 8.65
	F1-score	17.99 ± 25.98	54.52 ± 8.65	66.25 ± 8.80	72.43 ± 5.06
Set 7	ACC Global	50.36 ± 8.70	59.51 ± 5.99	68.65 ± 7.95	76.46 ± 6.51
	ATFL	68.92 ± 30.36	60.99 ± 12.56	72.57 ± 14.54	80.97 ± 9.90
	HealthyControl	32.15 ± 34.19	57.95 ± 10.00	64.82 ± 10.35	71.84 ± 7.67
	F1-score	53.64 ± 20.43	59.63 ± 7.93	69.31 ± 9.70	77.34 ± 6.88
Set 8	ACC Global	71.95 ± 6.83	68.41 ± 9.64	58.04 ± 6.00	78.78 ± 5.92
	ATFL	73.58 ± 9.03	67.66 ± 11.93	70.89 ± 29.58	80.19 ± 8.07
	HealthyControl	70.41 ± 10.63	69.21 ± 10.96	46.39 ± 26.99	77.44 ± 9.46
	F1-score	72.25 ± 6.58	67.88 ± 10.01	58.24 ± 20.06	78.96 ± 5.77
Set 9	ACC Global	65.12 ± 5.97	59.39 ± 5.41	58.04 ± 6.00	62.80 ± 4.21
	ATFL	69.96 ± 14.58	63.65 ± 9.00	70.89 ± 29.58	75.02 ± 12.15
	HealthyControl	60.27 ± 10.47	55.17 ± 9.08	46.39 ± 26.99	50.88 ± 13.31
	F1-score	65.89 ± 8.20	60.68 ± 5.89	58.24 ± 20.06	66.36 ± 4.80
Set 10	ACC Global	60.12 ± 6.78	57.92 ± 4.99	65.48 ± 6.13	64.63 ± 4.11
	ATFL	61.95 ± 14.54	59.32 ± 10.99	77.72 ± 12.80	69.15 ± 10.49
	HealthyControl	58.48 ± 9.81	56.55 ± 7.64	53.39 ± 12.50	60.21 ± 9.55
	F1-score	60.07 ± 10.43	58.13 ± 7.06	68.98 ± 6.47	65.87 ± 5.56
Set 11	ACC Global	49.39 ± 1.86	58.17 ± 6.95	62.31 ± 4.39	78.90 ± 5.93
	ATFL	9.25 ± 27.76	55.41 ± 9.15	69.30 ± 7.65	82.65 ± 8.35
	HealthyControl	90.00 ± 30.00	61.01 ± 13.41	55.30 ± 9.33	75.13 ± 7.91
	F1-score	6.21 ± 18.65	56.86 ± 6.71	64.68 ± 4.11	79.55 ± 6.11
Set 12	ACC Global	52.56 ± 5.89	60.85 ± 6.38	71.09 ± 7.03	80.60 ± 5.08
	ATFL	42.41 ± 43.44	59.57 ± 9.06	75.77 ± 12.73	**84.34 ± 6.79**
	HealthyControl	62.39 ± 42.59	62.23 ± 10.82	66.39 ± 9.33	76.83 ± 8.99
	F1-score	33.87 ± 31.76	60.48 ± 6.49	64.68 ± 4.11	81.48 ± 4.68
Set 13	ACC Global	50.60 ± 4.28	62.19 ± 5.94	70.36 ± 6.95	76.09 ± 6.43
	ATFL	68.67 ± 37.71	61.95 ± 9.41	75.64 ± 9.73	81.19 ± 8.34
	HealthyControl	33.40 ± 39.97	62.48 ± 9.89	65.30 ± 13.51	71.00 ± 7.47
	F1-score	50.97 ± 23.54	61.89 ± 6.58	71.79 ± 6.31	77.15 ± 6.33
Set 14	ACC Global	53.53 ± 7.99	60.24 ± 6.41	67.92 ± 5.57	82.56 ± 6.27
	ATFL	39.85 ± 40.03	60.75 ± 9.12	76.44 ± 6.62	83.85 ± 8.96
	HealthyControl	68.01 ± 38.75	59.72 ± 8.70	59.53 ± 10.73	81.36 ± 8.70
	F1-score	34.45 ± 27.98	60.11 ± 6.70	70.31 ± 4.53	82.62 ± 6.43
Set 15	ACC Global	56.09 ± 10.34	60.48 ± 6.96	72.43 ± 5.29	81.70 ± 5.94
	ATFL	49.59 ± 31.67	61.88 ± 12.69	78.58 ± 13.05	83.38 ± 11.25
	HealthyControl	63.27 ± 32.64	58.98 ± 12.01	66.35 ± 8.56	79.95 ± 9.35
	F1-score	46.76 ± 24.75	60.55 ± 8.49	73.54 ± 7.02	81.77 ± 6.47

**Table 6:** Training and testing times of the chosen algorithms on the ankle MRI images.

Algorithms	Metrics (%)	MLP	kNN	SVM	RF
Set 1	Training	0.01696	0.00026	7.99108	0.07679
	Test	0.00024	0.00110	0.00022	0.00659
Set 2	Training	0.78163	0.00019	0.00087	0.08279
	Test	0.00025	0.00109	**0.00019**	0.00672
Set 3	Training	0.01699	0.00031	0.00090	0.08290
	Test	0.00028	0.00115	0.00024	0.00718
Set 4	Training	1.01803	0.00031	0.13495	0.07867
	Test	0.00025	0.00115	0.00022	0.00683
Set 5	Training	0.03747	0.00020	3.61693	0.07941
	Test	0.00023	0.00099	0.00023	0.00672
Set 6	Training	0.02615	0.00030	3.67280	0.08182
	Test	0.00026	0.00112	0.00024	0.00696
Set 7	Training	0.03339	0.00029	13.56860	0.08361
	Test	0.00026	0.00121	0.00021	0.00690
Set 8	Training	0.90728	0.00020	0.00093	0.08563
	Test	0.00022	0.00108	0.00021	0.00696
Set 9	Training	0.77169	0.00021	0.14240	0.08291
	Test	0.00029	0.00107	0.00024	0.00679
Set 10	Training	1.41660	0.00028	0.12939	0.08386
	Test	0.00027	0.00121	0.00023	0.00743
Set 11	Training	0.02186	0.00020	3.24591	0.08495
	Test	0.00023	0.00109	0.00023	0.00688
Set 12	Training	0.03380	0.00019	11.99871	0.08607
	Test	0.00027	0.00118	0.00022	0.00666
Set 13	Training	0.03712	**0.00018**	13.16548	0.07790
	Test	0.00024	0.00105	0.00023	0.00662
Set 14	Training	0.04402	0.00021	14.03406	0.08386
	Test	0.00027	0.00105	0.00023	0.00665
Set 15	Training	0.08085	0.00022	12.39197	0.08032
	Test	0.00029	0.00106	0.00023	0.00638

## Data Availability

Not applicable.
